# Mechanisms and Impact of Cognitive Reserve in Normal Aging and Alzheimer’s Disease

**DOI:** 10.3390/diagnostics15233068

**Published:** 2025-12-02

**Authors:** Chanda Simfukwe, Seong Soo A. An, Young Chul Youn

**Affiliations:** 1 Department of Bionano Technology, Gachon University, 1342 Seongnam-daero, Seongnam-si 13120, Republic of Korea; chandaelizabeth94@gmail.com; 2 Department of Neurology, College of Medicine, Chung-Ang University, Seoul 06974, Republic of Korea; 3 Department of Medical Informatics, Chung-Ang University College of Medicine, Seoul 06974, Republic of Korea

**Keywords:** cognitive reserve, Alzheimer’s disease, mild cognitive impairment, aging, biomarkers

## Abstract

Age-related cognitive decline and individual differences in dementia susceptibility are increasingly explained through the concept of cognitive reserve (CR). CR reflected the brain’s adaptive capacity to sustain cognitive performance despite Alzheimer’s disease (AD)-related pathology, extending beyond traditional biomarkers that captured the molecular or structural changes, but often failed to account for clinical heterogeneity. This review provided a comprehensive synthesis of how CR was operationalized through three major methodological approaches: sociobehavioral proxies, residual variance frameworks, and neurobiological indicators within the context of longitudinal study designs. The review included evidences from a structured PubMed and Scopus search restricted to English-language studies examining the incidence of mild cognitive impairment (MCI) or AD. Findings consistently demonstrated that higher CR, most commonly estimated through sociobehavioral proxies, such as educational level, occupational complexity, bilingualism, and engagement in cognitively stimulating activities, was associated with a delayed onset of impairment, lower dementia risk, and better clinical outcomes, despite a comparable neuropathological burden. Residual variance approaches provided complementary insights by quantifying cognitive performance that exceeded the predicted levels from underlying pathology, thereby capturing unexplained variance by structural or molecular disease markers. These residual-based methods extend CR concept beyond life-course experiences, offering statistical evidence of resilience within longitudinal trajectories of aging and disease. Additional evidence from electrophysiological and genetic investigations further suggested that CR enhanced the neural efficiency, flexibility, and the recruitment of compensatory networks. Finally, neuroimaging studies provided the mechanistic evidence that CR was supported by alterations in brain structure, functional connectivity, and activation patterns, though findings on long-term trajectories remained inconsistent. Overall, CR emerged as a multidimensional and modifiable construct that enhanced resilience to aging and dementia. Future research should prioritize the integrative longitudinal designs, combining sociobehavioral, residual variance, genetic, electrophysiological, and neuroimaging approaches to clarify mechanisms, establishing robust measurement frameworks and advance clinical translation.

## 1. Introduction

Increasing global life expectancy resulted in a rapid expansion of the population aged 65 years and older with a concomitant rise in the prevalence of projected dementia over the coming decades [1]. Alzheimer’s disease (AD) constituted the most prevalent cause of dementia, and its worldwide prevalence was anticipated to nearly double within the next 20 years [1,2]. The dementia continuum encompassed the distinct clinical stages, beginning with subjective cognitive decline (SCD), a self-reported perception of cognitive worsening in the absence of measurable deficits on standardized neuropsychological assessments, and progressing to mild cognitive impairment (MCI), in which objective deficits were detectable, but the functional independence was maintained [3,4,5]. At the advanced stage, AD was marked by progressive and irreversible cognitive deterioration that ultimately disrupted the independent daily functioning [6,7]. Both SCD and MCI were recognized as prodromal states that conferred a substantially elevated risk for subsequent progression to AD [3,5].

The pathological hallmarks of AD included the accumulation of β-amyloid (Aβ) plaques and tau-related neurofibrillary tangles, both of which could be quantified through advanced neuroimaging modalities and fluid-based biomarker assays [8,9,10]. The National Institute on Aging-Alzheimer’s Association (NIA-AA) Research Framework formalized the AT(N) classification, Aβ deposition (A), tau pathology (T), and neurodegeneration (N), as the core biological indicators of AD presence and severity [11]. These biomarkers provided an important framework for staging disease progression and differentiating preclinical, prodromal, and dementia phases, thereby enabling a more biologically grounded understanding of AD. In the absence of disease-modifying therapies, identifying factors that could delay or attenuate progression to dementia remained a priority, given the profound implications for public health expenditure and the preservation of quality of life [11,12,13]. Despite considerable scientific progress and significant investments in drug discovery, the multifactorial complexity of AD pathophysiology was thus far impeded the development of effective therapeutics, emphasizing the need for innovative treatment strategies [14].

In this context, growing research attention was shifted toward protective and resilience mechanisms, which might explain why some individuals maintained cognitive function despite substantial neuropathological accumulations [15,16]. It was increasingly recognized that the degree of cognitive impairment did not always correspond directly to neuropathological burden; individuals with comparable levels of amyloid accumulations or brain atrophy may exhibit markedly different clinical trajectories [17,18]. This dissociation was attributed to interindividual variability in resilience against AD-related pathology, conceptualized as cognitive reserve (CR) [15,19].

### Cognitive Reserve

CR may be one mechanism through which individuals were protected against significant clinical cognitive decline, even in the presence of neuropathology [15,19,20]. The concept was based on the notion that sociobehavioral proxies, such as education, intellectually engaging occupations, and various other activities contributed to the development of resilient neuronal networks that helped to preserve cognitive function, even as AD biomarkers indicated progressing pathology [19,20,21]. Moreover, CR was further expected to moderated the association between neuropathology and cognitive performance; that was, individuals with higher CR demonstrated the greater resilience against AD-related neuropathological burden [22].

Originally proposed to explain discrepancies between brain pathology and clinical manifestation, CR framework now encompassed the neural efficiency, network flexibility, and compensatory activation, as potential mechanisms that sustain performance [23,24]. Subsequent research identified three primary approaches to operationalizing CR. The first and most widely applied approach relied on sociobehavioral proxies, such as educational attainment, occupational complexity, bilingualism, and engagement in cognitively stimulating activities, as indirect indicators of reserve [19,23,24]. The second approach utilized residual variance methods, in which CR was quantified as the discrepancy between observed cognitive performance and performance, predicted from underlying neuropathology, thereby capturing unexplained variance by structural or molecular disease markers [19]. The third and emerging approach seeked to move beyond proxy and residual frameworks by incorporating neuroimaging, electrophysiological, and genetic indicators to provided more direct and mechanistic assessments of CR [22,25,26,27]. Convergent evidence across these approaches indicated that higher levels of CR were associated with delayed onset of cognitive impairment and reduced risk of dementia progression, irrespective of AD-related pathology [15,19,28]. Accordingly, CR offered a unifying explanatory model that associated life-course enrichment, neural adaptability, and disease compensation to observed variability in cognitive aging trajectories.

## 2. Conceptual Framework and Hypothesized Effects of Cognitive Reserve

The concept of CR proposed that higher levels of reserve influenced both cognitive and clinical trajectories across aging and disease [15]. Stern’s [19] model suggested that individuals with greater CR, not only achieved superior cognitive performance before the onset of decline, but also experienced a delayed emergence of disease-related impairment. At the same time, because a greater reserve allowed individuals to tolerate more extensive neuropathology before symptoms manifest, once pathology surpassed a critical threshold, decline may progress more rapidly [29]. Although developed primarily to account for AD, this framework may also explain variability in cognitive outcomes associated with other neuropathologies and age-related brain changes [30].

CR was a theoretical construct that could not be directly observed and was therefore most commonly inferred through indirect measures [19]. The most widely used operationalization relied on sociobehavioral proxies that reflected the lifetime experiences, including educational level, occupational complexity, bilingualism, and engagement in cognitively stimulating activities [15,31]. These variables were not mutually exclusive and often overlap; for example, individuals from higher socioeconomic status backgrounds were more likely to achieve higher education, which may lead to greater occupational attainment, higher income, and access to enriching leisure activities [32].

To strengthen the conceptual foundation of this review, it was important to distinguish CR from related constructs that were often conflated in the literature: brain reserve, compensation, and reserve capacity. CR referred to adaptive functional processes, such as neural efficiency, flexible strategy use, and compensatory network recruitment, that helped to maintain performance despite pathology [15,19]. Brain reserve denoted passive structural endowments, including brain volume, synaptic density, and neuronal count, that buffered the initial impact of neuropathology [19,32]. Compensation referred to the observed active neural adjustments during task engagement, such as the recruitment of additional or alternative networks [23,30]. Reserve capacity reflected the upper limit of adaptive potential that could be mobilized, as pathology progresses [30]. Importantly, residual variance approaches indexed the functional compensation, rather than CR capacity, as they captured the performance exceeding levels from the predicted biomarkers or structural integrity [33].

Beyond proxies, residual variance approaches estimated CR statistically, as the discrepancy between the observed cognitive performance and predicted performance from levels of neuropathology, thereby capturing the unexplained variance by structural or molecular markers [33,34]. In addition, neurobiological indicators, including neuroimaging, electrophysiological, and genetic measures, offered better direct mechanistic assessments of reserve capacity [19,22]. Ongoing research sought to delineate the relative contributions of proxy-based, residual, and neurobiological indicators of cognitive reserve to trajectories of cognitive aging and dementia risk.

In parallel with CR, contemporary consensus frameworks emphasized the related construct of brain maintenance, defined as the relative absence or slowing of age- and disease-related changes in neural resources or neuropathology over time [19]. Brain maintenance was typically indexed by longitudinal preservation of brain structure and microstructural integrity (e.g., cortical thickness, white matter diffusivity), whereas CR referred to the functional adaptability that allowed individuals to perform better than expected given their level of brain change or injury [19,23,30]. Recent longitudinal work illustrated this distinction: Gazes et al. showed that the preserved cortical thickness and lower white matter mean diffusivity were associated with the reduced five-year decline in multiple cognitive abilities (consistent with brain maintenance), while a higher intelligence quotient (IQ) and education independently buffered the decline after accounting for structural change, consistent with CR mechanisms [35]. These findings focused that brain maintenance reduced the accumulation of damage, whereas CR moderated the impact of existing damage on behavioral performance, and that both processes made the partially dissociable contributions to cognitive aging trajectories.

## 3. Purpose of Current Review

**Figure 1:**
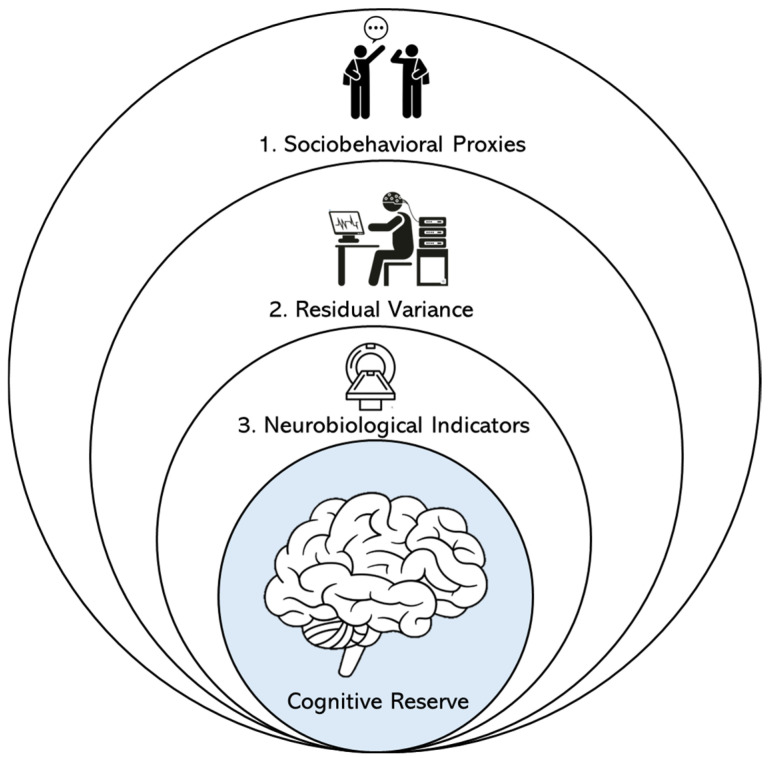
The figure illustrates three approaches to operationalizing CR, represented as concentric layers surrounding the core construct. (1) Sociobehavioral proxies serve as indirect indicators, (2) residual variance methods capture cognitive performance beyond pathology-predicted levels, and (3) neurobiological indicators provided direct mechanistic measures. Abbreviations: CR, Cognitive Reserve.

Studies consistently demonstrated that individuals with higher CR could withstand greater levels of AD-related neuropathology before manifesting significant cognitive decline or clinical impairment [15,19,22,26]. This protective effect positioned CR, as a key explanatory construct, for interindividual variability in dementia susceptibility and progression. However, much of the literature on incident dementia relied predominantly on sociobehavioral proxies of CR, such as educational attainment or occupational complexity without consistently integrating direct measures of neuropathology [19,36,37]. This limitation restricted the rigorous testing of CR hypothesis and reduced the explanatory power for heterogeneous clinical outcomes. Building on prior work, this review systematically synthesized the longitudinal cohort studies that examined the role of CR in the incidence of MCI and AD [22,36,38]. Specifically, this review study centered on three complementary methodological approaches to operationalizing CR (Figure 1): (1) sociobehavioral proxies, (2) residual variance frameworks, and (3) neurobiological indicators. Drawing on evidence from longitudinal cohort studies, how each approach contributed to understanding the mechanisms through which CR was critically evaluated and moderated the relationship between AD-related neuropathology and cognitive outcomes. By synthesizing findings across these domains, the review provided a multidimensional assessment of CR, as both a statistical and neurobiological construct of resilience. The review further delineated the persistent methodological limitations, including the predominant reliance on proxy-based measures and cross-sectional designs, and articulated research priorities that emphasized the need for integrative longitudinal models combining sociobehavioral, residual, genetic, electrophysiological, and neuroimaging approaches to elucidate mechanisms, refined measurement frameworks, and facilitated clinical translation.

Table 1 provided a concise comparison of representative longitudinal and biomarker-based CR studies and made points in several methodological issues that constrained the interpretation across the literature. Although many studies controlled for demographics and AD biomarkers, key confounders, particularly SES, education quality, and APOE ε4 genotype, were inconsistently measured or omitted, increasing the risk of social and genetic confounding. The table also illustrated substantial heterogeneity in CR operationalization, ranging from education and IQ to residual-variance indices and network-based neuroimaging metrics, which contributed to variability in reported effect sizes and trajectories. Notably, studies with multimodal biomarker adjustment or network-level measures tended to provided better stable estimates of CR, whereas those lacking SES or APOE control were difficult to interpret. Overall, this comparison emphasized the need for greater methodological consistency, better comprehensive confounder adjustment, and harmonized CR definitions to strengthen causal inference in future research.

## 4. Literature Search

**Figure 2:**
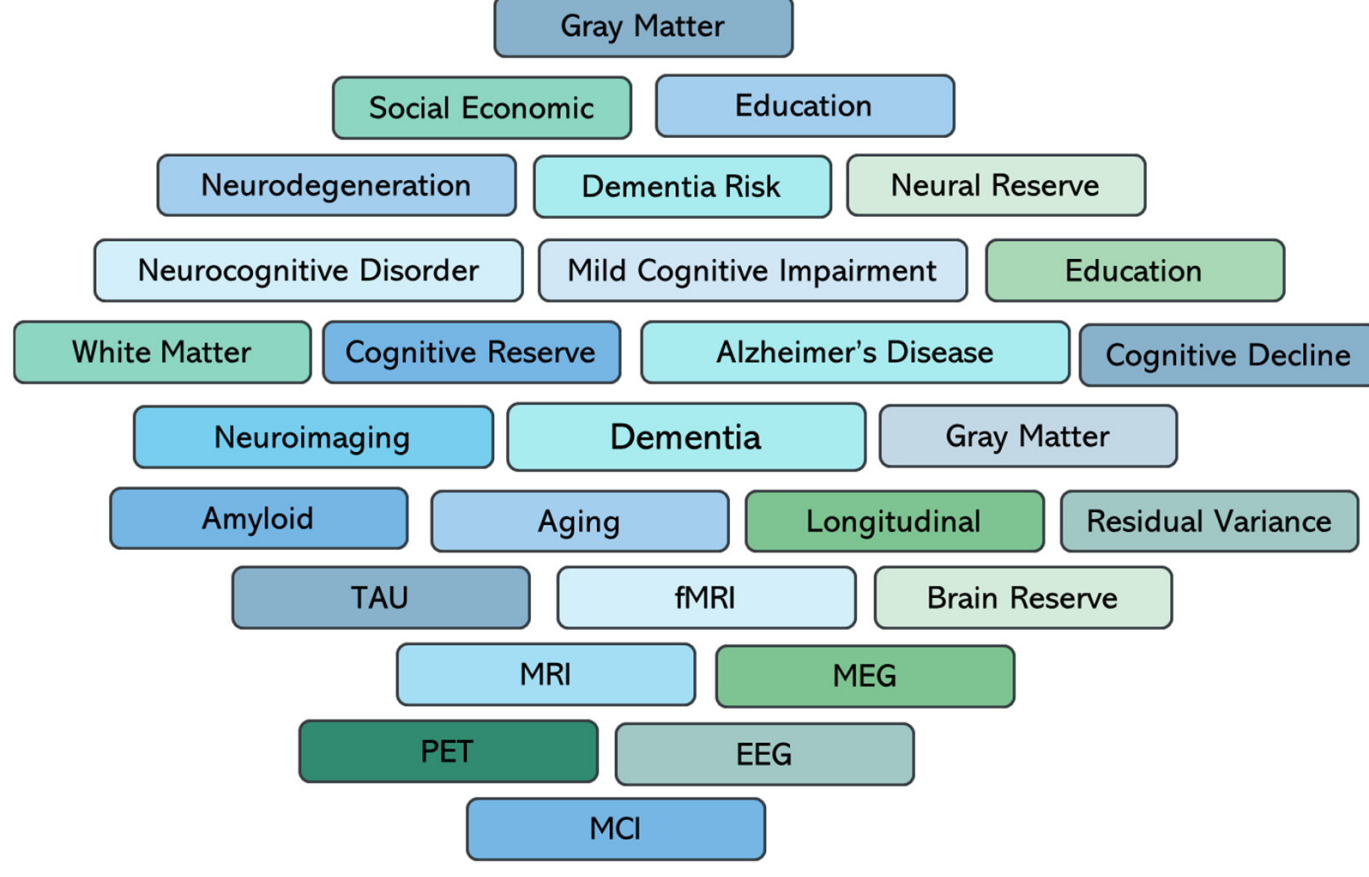
Search terms applied in the structured literature search across PubMed, Scopus, and Google Scholar. The visualization illustrated the core domains considered in this review, including cognitive reserve, dementia risk and progression, biomarkers, and cognitive outcomes. These keywords were used to guide the identification of eligible longitudinal studies published. Abbreviations: TAU, phosphorylated tau (p-tau); fMRI, functional magnetic resonance imaging; MRI, magnetic resonance imaging; MEG, magnetoencephalography; PET, positron emission tomography; EEG, electroencephalography; MCI, mild cognitive impairment.

To support this narrative review, a structured literature searches of PubMed and Scopus were conducted and supplemented by a review of Google Scholar to capture additional relevant records. Search strategies combined both natural language terms and controlled vocabulary (MeSH in PubMed) with Boolean operators (AND, OR) applied to refine queries. Four conceptual domains were targeted: (1) cognitive reserve, (2) dementia progression and neuropathology, (3) biomarkers of disease burden, and (4) cognitive outcomes. Search terms were selected to capture the breadth of concepts relevant to cognitive reserve, dementia progression, biomarkers, and cognitive outcomes. The complete set of keywords applied in the structured literature searches was illustrated in Figure 2, which summarized the domains and terms guiding this review. These terms were selected to capture the breadth of CR operationalizations, spanning sociobehavioral proxies, residual variance methods, and biomarker-based measures, derived from neuroimaging, electrophysiology, and genetics. Eligible studies were restricted to longitudinal cohort designs, published in English, and explicitly investigating the incidence or progression of MCI or dementia. Exclusion criteria included cross-sectional studies, case reports, conference abstracts, and non-peer-reviewed publications. The search covered all records available in PubMed and Scopus from database inception through August 2025 and was supplemented by targeted Google Scholar screening. Grey literature sources were not included, and databases, such as PsycINFO and Web of Science, were not part of the structured search. This review was based entirely on previously published data and did not involve any original research with human or animal participants.

## 5. Sociobehavioral Proxy Measures of Cognitive Reserve

Studies using longitudinal and epidemiological cohorts provided a unique approach for investigating how CR affects long-term clinical and cognitive outcomes, particularly the risk of dementia [38,42]. The most widely used proxy measure of CR in this body of evidence was the years of formal education. Several studies showed that a greater level of education was associated to a lower risk of dementia and incident MCI [38,42,43,44]. However, these correlations were not consistently found in all studies, and the results often differed depending on the operationalization of education [45,46]. Although it was simple to measure, years of education were a static indicator that did not account for cognitive engagement throughout one’s life and did not accurately reflect the quality and variety of learning experiences [15]. As a result, various proxies, such as literacy, reading ability, and vocabulary, were suggested as more reliable markers of reserve [47,48]. Evidence suggested that literacy, reading, and vocabulary assessments tended to exhibit stronger correlations with the risk of MCI or dementia than the number of years of education [47,48,49,50].

Accumulating evidence suggested that socioeconomic and occupational factors played a protective role against dementia [24]. Individuals with higher occupational attainment or greater work complexity tended to exhibit a reduced risk of dementia [50,51,52]. Cognitively demanding activities, particularly those that required information processing and pattern recognition, appeared to be especially beneficial [53,54]. Later retirement was likewise associated with lower dementia incidence, emphasizing the value of prolonged cognitive engagement across the life-course [55]. In parallel, markers of socioeconomic status, including elevated household income and wealth, were consistently associated to the reduced vulnerability to dementia [50,53]. Engagement in leisure pursuits that were cognitively, socially, and physically stimulating was consistently associated to a lower risk of both MCI and dementia [50,51,52,53,54]. Activities, such as reading, playing games, attending museums or concerts, volunteering, and making music, were all associated with protective effects [50,53,55].

Fratiglioni et al. [56] emphasized that the social, cognitive, and physical dimensions of lifestyle, each contributed to reducing the risk of dementia. Importantly, many of these activities were multidimensional in nature. For instance, social participation often provided cognitive stimulation, while group-based physical activities, such as aerobics or tai chi, combine physical exercise embraced the social and mental engagement [57,58,59]. Rather than the specific type of activity undertaken, several studies suggested that the diversity and breadth of engagement may be particularly critical for reducing dementia vulnerability [60].

Experiences and abilities established early in life were shown to influence the likelihood of developing cognitive impairment in later years with some associations persisting even after accounting for adult educational or occupational achievements [61,62]. Evidence suggested that stronger childhood academic performance, higher cognitive test scores at age 11, greater socioeconomic resources during childhood, and more complex written expression by early adulthood were all associated with a reduced risk of dementia [63,64,65]. In contrast, exposure to early adversity, such as parental loss, was associated with a higher prevalence of Alzheimer’s dementia [66,67]. Developmental factors before birth may also shape late-life vulnerability; lower birth weight and smaller head circumference were both implicated as potential risk markers for dementia [68]. Findings on bilingualism, however, remained inconsistent. While some reports suggested protective effects, a recent meta-analysis concluded that bilingualism did not significantly reduce the risk of cognitive decline or dementia [69,70].

Collectively, the evidence indicated that the elevated performance on CR proxy measures was consistently associated with a reduced risk of MCI and dementia [32,33]. If one assumed that neuropathological burden accrued at a comparable rate across individuals, these findings indirectly suggested that persons with higher levels of CR could tolerate greater neuropathology before clinical symptoms or functional decline emerge [19,22,29]. This interpretation aligned closely with the reserve framework outlined in prior theoretical models [15].

### Social Cognitive Reserve Proxies and Cognitive Decline in Cohort Studies

Previous peer-reviewed publications demonstrated that higher levels of CR, operationalized through proxy indicators, were strongly correlated to better cognitive performance in midlife and older adulthood [71,72]. Commonly used proxies included years of formal education, occupational attainment, and socioeconomic status, as well as participation in cognitively, socially, and physically enriching leisure activities [51,52,53,54,55,56,57,58,59,60,61,71,72]. According to Stern’s model of CR, individuals with greater reserve consistently outperformed those with lower reserve, as aging and neuropathological processes advance [19]. The framework further positioned that higher CR enabled individuals to tolerate greater levels of pathology before manifesting clinical symptoms, thereby delaying the emergence of cognitive and functional decline [15,19,22]. Several longitudinal studies revealed that higher CR was associated with a postponed onset of MCI, dementia, and measurable cognitive decline [29,73,74].

Findings on the influence of CR on longitudinal patterns of cognitive change remained inconsistent. Some investigations demonstrated that higher CR was associated with slower rates of decline, whereas others reported steeper declines among individuals with greater CR, at least on specific cognitive measures [15,22,32,73,75]. Still other studies observed the initial differences in baseline cognitive performance by CR level, yet found no significant variation in subsequent trajectories of decline [76,77,78]. Multiple influences, including cohort characteristics, methodological approaches, and measurement strategies, likely shaped discrepancies in the literature regarding CR and longitudinal clinical or cognitive outcomes. A notable limitation of many studies was the inclusion of participants, whom were non-demented at baseline, a group often comprising both cognitively normal individuals and those with MCI. These subgroups differed in important respects, such as baseline cognitive ability, levels of CR, and extent of underlying neuropathology, which may confound the subsequent cognitive and clinical trajectories [47,48,49,55].

Evidence from studies that stratified by clinical impairment at baseline or the follow-up suggested a nuanced pattern. In individuals, whom progressed to MCI or dementia, higher CR was frequently noticeable with steeper rates of cognitive decline, once clinical symptoms emerged [15,19,79,80], consistent with Stern’s reserve framework [19]. In contrast, among older adults, who did not have dementia, CR appeared to have a lesser influence on the rate of decline. Instead, its effect may manifest through maintaining higher levels of performance, despite gradual accumulation of pathology, thereby delaying the threshold at which impairment became clinically evident [36,73]. Variability across studies may also reflect differences in demographic composition and study design, including participants’ baseline age and the duration of longitudinal follow-up. A more critical synthesis suggested that heterogeneity in post-onset decline may reflect several interacting moderator variables, rather than true contradictions in the literature. Differences in underlying pathology burden appeared to be central; individuals with high CR may show slower decline when amyloid/tau levels or atrophy rates were modest, but steeper decline once neuropathology exceeded a compensatory threshold, a phenomenon consistent with the “CR Overload” or “threshold” model [81]. Follow-up duration also contributed to inconsistent findings, as short intervals may predominantly captured the pre-threshold stability in high-CR individuals, whereas longer follow-up periods were more likely to detect the accelerated decline that occurred once compensatory mechanisms failed [82]. Variability in cohort characteristics, including baseline diagnostic status and age, further moderated trajectories by influencing both the onset and timing of compensatory exhaustion [83,84]. Considering these moderators provided a better coherent explanatory account for observed divergent longitudinal patterns across studies. 

**Figure 3:**
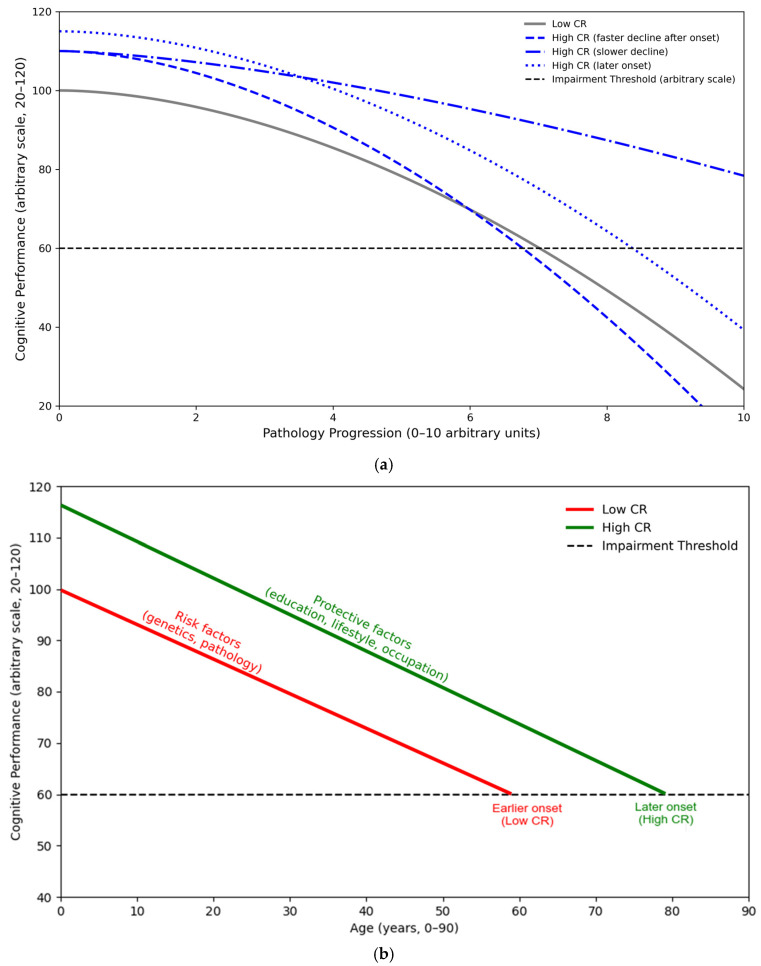
Conceptual models illustrating how CR modified the association between cognitive performance, pathology progression, and age. (**a**) pathology-based model; the curve showed hypothetical trajectories of Cognitive Performance (*y*-axis, arbitrary scale 20–120) against Pathology Progression (*x*-axis, 0–10 units). Individuals with low CR (gray line) exhibited an earlier and smoother decline, while those with high CR (blue lines) maintained higher cognitive function and reached the impairment threshold (dashed line = 60) later. High CR may present as a later onset of decline, a slower rate of deterioration, or, once compensatory mechanisms were exceeded, a steeper post-onset decline [15,19,22]. These variations indicated that observed differences across studies may depend on which segment of the cognitive trajectory was captured. (**b**) lifespan-based model; the trajectories plot Cognitive Performance (*y*-axis, 20–120) against age (years, 0–90). The high-CR line (green) remained the elevated relative to the low-CR line (red), demonstrating how protective factors, such as education, complex occupations, and cognitively stimulating lifestyles, preserve function and delay the onset of impairment. In contrast, risk factors, such as genetic susceptibility, neuropathology, and poor health accelerate, declined and led to the emergence of symptoms at an earlier stage [49,53,54,55,56,64]. Note: Early onset indicated that cognitive decline began at a younger age or earlier stage of pathology, whereas late onset reflected a delayed emergence of impairment due to higher cognitive reserve and greater resilience to brain changes. Abbreviations: CR, Cognitive Reserve.

Cohorts recruited in midlife often required an extended observation periods before measurable cognitive decline emerges, whereas studies involving older populations may be influenced by selective survival effects that attenuated the observed associations [61,75]. Figure 3a,b illustrated these conceptual trajectories, demonstrating how cognitive reserve modulated the relationship between pathology progression, age, and performance, thereby delaying impairment onset and shaping post-onset decline patterns.

Methodological constraints likely contributed to the inconsistencies reported across studies [15,19,23,28,31,46,77]. As indicated in prior reviews, several early investigations suffered from statistical limitations that may have biased their conclusions [32,43]. Only a small number of studies [71,78] were sufficiently powered to assess the impact of very low levels of CR, which restricted the applicability of findings to broader populations. This limitation may reflect both methodological choices and sample characteristics [77]. Variation in how CR was measured and operationalized also presented a significant source of inconsistency [80]. Studies often differed in the selection, collection, and analytical treatment of proxy variables, which complicated cross-study comparisons and potentially obscures true associations [85,86]. Lastly, epidemiologic research on cognitive reserve was typically constrained by insufficient measures of underlying pathology or age-related brain alterations [16]. Consequently, these studies were unable to directly assess the impact of CR on the relationship between age- and disease-related brain changes and cognitive performance, nor did they elucidate the mechanisms that underlied CR [87].

Growing evidence indicated that cognitive reserve was not only shaped by lifetime sociobehavioral experiences, but could also be enhanced through targeted interventions in later life. Recent multidomain trials by Marselli et al. [88] demonstrated that structured cognitive training, when combined with physical activity and lifestyle modifications, could enhance cognitive performance and potentially slowed the progression toward MCI or dementia. These findings indicated the modifiable nature of CR and reinforced the potential of preventive strategies that promoted the sustained cognitive engagement, social participation, and healthy behaviors. When interpreting CR proxies, it was also essential to consider cultural, socioeconomic, and educational contexts, as years of schooling, literacy, occupational complexity, and leisure opportunities varied substantially across global populations. Differences in education quality, informal learning environments, and linguistic or cultural practices could influence both the validity and generalizability of CR measures. Incorporating culturally sensitive indicators and adjusting for educational quality will therefore enhance the accuracy of CR estimation and improve cross-population comparability.

## 6. Residual Variance Approaches to Cognitive Reserve

Residual variance methods estimated CR using a statistical modeling framework that isolated the portion of cognitive performance, not explained by brain structure, pathology, or demographic factors [76,89]. In this approach, researchers constructed the regression or structural equation models, in which cognitive outcomes, commonly episodic memory, executive function, or global cognition, were predicted from brain integrity markers, such as hippocampal volume, cortical thickness, amyloid or tau burden, and white matter hyperintensities, together with covariates like age, sex, and education [87,89]. The residuals, defined as the difference between observed and predicted cognitive scores, represented the “better-than-expected” performance given an individual’s neural status and demographic profile [19,86]. These residuals were interpreted as an empirical index of reserve capacity, reflecting latent resilience mechanisms that enabled the maintained cognitive function, despite equivalent neuropathological load [78,87].

Statistically, this method captured interindividual variability that remained, after accounting for known structural or molecular determinants of cognition [88,90]. Unlike proxy measured that relied on life experience indicators (e.g., education or occupation), the residual variance approach quantified CR directly within the data-generating model, providing an individualized and continuous estimate of reserve [87]. Cross-sectional studies employing this framework consistently reported that individuals with higher residual-based CR exhibited superior performance across multiple domains, despite comparable brain atrophy or amyloid deposition [73,87,91]. Reed et al. [78] demonstrated that residual memory variance predicted the global cognitive outcomes independently of gray matter loss, while subsequent work confirmed that higher residual CR was associated with preserved executive and global functioning in both MCI and AD cohorts.

Nevertheless, the residual approach presented important methodological considerations. Because residuals were mathematically derived from cognitive scores, they may remain correlated with the original cognitive variable and could absorb unexplained measurement error or unmeasured neural influences [40]. The validity of residual-based CR indices depended critically on the completeness and reliability of the predictor set. The omission of key neuropathological or neurobiological variables may distort residual estimates, thereby conflating measurement error with genuine reserve effects [19,40,87,91].

### Longitudinal Evidence for Residual-Based Cognitive Reserve

Longitudinal applications of residual variance models extended this framework by examining how “better-than-expected” cognitive performance predicted future trajectories of decline. In these studies, linear mixed-effects models and latent growth-curve analyses were typically employed to estimate within-individual change in cognition over time, while residual-based CR was entered, as a moderator or predictor of slope and intercept parameters [34,39,92]. Zahodne et al. [89] applied a mixed-effects modeling to demonstrated that higher residual memory variance at baseline predicted a slower rate of global cognitive decline over three years, supporting the interpretation of CR, as a dynamic resilience factor rather than a static trait. Similarly, Soldan et al. [39] and Gallo et al. [34] demonstrated that residual-based CR moderated the longitudinal relationship between brain integrity and cognition, attenuating the impact of cortical thinning and hippocampal atrophy on cognitive decline and lowering the likelihood of conversion from normal cognition to MCI or dementia. More recent research, as reported by Levin et al. [93], integrated the residual-derived CR within multimodal longitudinal frameworks, revealing that reserve interacted with metabolic and structural Alzheimer’s subtypes, suggesting heterogeneous compensatory mechanisms across disease phenotypes. Despite these advances, longitudinal research remained to be constrained by short follow-up durations and reliance on single-domain residuals, most often derived from episodic memory tasks.

A more critical view of the residual-variance framework revealed methodological challenges that limited its validity as an indicator of CR. Because residuals were mathematically tied to both the cognitive outcome and the predictor set, they inevitably incorporated measurement error, fluctuations in non-specific cognitive states (e.g., fatigue, alertness, mood), and the influence of unmeasured confounding variables [94]. When key biomarkers, such as vascular, metabolic, or network-level measures, were omitted, residual estimates may be artificially inflated by variance attributable to these missing factors rather than by reserve itself. Consequently, residual-based indices often represented a composite of compensatory processes, noise, and unmeasured pathology rather than a direct estimate of CR capacity. Recognizing these statistical and interpretive constraints was essential, when applying or comparing residual-variance approaches in studies of cognitive aging and dementia. Future studies should implement time-varying residuals, multidomain cognitive composites, and biomarker-coupled longitudinal models to capture the evolving nature of reserve as pathology progresses.

## 7. Neurobiological Indicator Approaches to Cognitive Reserve

Most biomarker studies examining CR were conducted in cross-sectional designs [19,27]. Across both cognitively unimpaired individuals and those with MCI or dementia, findings consistently indicate that CR, typically operationalized through proxy measures, modifies the association between cognition or clinical outcomes and markers of neuropathology [39]. Evidence demonstrated such moderating effects across multiple modalities, including amyloid deposition, tau pathology, structural atrophy assessed with MRI, white matter hyperintensities (WMH), glucose metabolism measured with fluorodeoxyglucose positron emission tomography (FDG-PET), and cerebral perfusion [94,95,96,97]. These observations suggested that age- and disease-related brain alterations had a weaker effect on cognitive performance in individuals with higher CR. Nevertheless, results among cognitively normal populations were less consistent from several studies reporting better equivocal evidence of moderation [95,97].

Cross-sectional evidence also lends substantial support to the view that proxy indicators of CR correspond with markers of neural and brain reserve. Reported associations span multiple domains, including neural efficiency and capacity, structural indices such as global brain volume and white matter integrity, neurotransmission processes, and measures of cerebrovascular health [98,99,100].

Extending this work to large-scale population neuroimaging, DeJong et al. [41] demonstrated that white matter structural connectivity was an important substrate of cognitive resilience in middle-aged adults. In a cohort of nearly 4800 participants from the Maastricht study, greater global node degree attenuated the negative associations between brain atrophy, cerebral small vessel disease markers, and composite cognitive performance, such that individuals with high damage but dense connectivity networks exhibited near-normal cognitive performance. These findings support a “neural reserve” perspective in which the organization and robustness of large-scale structural networks confer resilience to vascular and neurodegenerative brain changes, complementing proxy-based and residual-variance approaches by providing a biologically grounded indicator of reserve capacity [41].

Functional MRI studies provided illustrative examples: individuals with higher CR appeared capable of compensating for age- or disease-related neural alterations by recruiting alternative mechanisms in response to cognitive demands [22,100]. Collectively, such findings elucidate the neurobiological mechanisms underlying CR and suggested potential pathways through which brain reserve may be enhanced [27,96,97,98]. Nevertheless, the evidence leading CR proxies directly to the degree of disease-related pathology remains mixed and inconclusive [96]. Cross-sectional biomarker studies were limited as they did not permit inferences regarding the direction of causality among the relationships between cognitive reserve, brain integrity, and cognition [95,96]. Furthermore, they did not facilitate an assessment of how proxy measures of cognitive reserve influence cognitive decline and clinical impairment during neuropathological and age-related brain alterations, nor did they clarify their direct effects on the rates of change in biomarkers over time.

A more integrated neurobiological framework suggested that neural efficiency and neural compensation may operate at different stages of AD pathology rather than functioning as interchangeable mechanisms. In preclinical or early-pathology stages, individuals with higher CR may exhibit greater neural efficiency, characterized by reduced activation in task-relevant regions for equivalent performance, reflecting optimized network utilization [15]. As pathology progresses, efficiency alone becomes insufficient, and individuals increasingly rely on compensatory network recruitment, including upregulation of frontoparietal control regions or bilateral activation patterns [15,23]. In later stages, as neurodegeneration intensifies and network integrity deteriorates, compensatory mechanisms may reach their limits, resulting in “compensation failure” and accelerated cognitive decline despite high CR [15,28,35]. This stage-dependent trajectory provided a more systematic conceptual model for interpreting heterogeneous neuroimaging findings and clarifies how CR-related mechanisms evolve across the AD continuum.

### Longitudinal Studies of Neurobiological Indicators

Relatively few longitudinal investigations were explored how CR interacts with AD biomarkers to influence cognitive and clinical trajectories. Evidence synthesized by Soldan et al. [73] suggested that the protective role of CR in delaying the progression from normal cognition to MCI was mainly independent of amyloid burden, as measured by cerebrospinal fluid (CSF) Aβ levels. Instead, CR and amyloid contribute additively to risk reduction. By contrast, some studies suggested that the protective effects of CR diminish as markers of neuronal injury, such as CSF total tau and MRI-based atrophy, become more pronounced [92]. However, this pattern suggested that the mechanisms underlying CR’s benefits may lose efficacy as neurodegeneration advances, or that these compensatory processes progressively deteriorate with disease progression [25]. Among individuals with MCI, studies indicate that for a given degree of cortical thinning, those with higher educational attainment experience a longer dementia-free interval compared to those with lower educational attainment [93]. Additional evidence suggested that education may also mitigate the detrimental effects of WMH on the risk of MCI and dementia [25]. In contrast, late-life engagement in leisure activities did not show to moderated the association between AD biomarkers and progression to dementia in non-demented cohorts [99].

In cognitively normal populations or those without dementia, evidence suggested that the beneficial effects of CR on cognitive performance were mainly independent of baseline AD biomarkers and cerebrovascular pathology [21,89,92]. However, it remains uncertain to what extent CR proxy measures moderated the relationship between biomarker burden and subsequent rates of cognitive decline from effects likely influenced by both clinical status and severity of pathology [100]. Previous study reported that higher CR was associated to accelerated decline following symptom onset among individuals who later progressed to MCI or dementia but showed no effect on trajectories in those who remained cognitively normal, regardless of baseline biomarker levels [21]. Similarly, across the AD spectrum, higher educational attainment was associated with equivalent or slower decline when atrophy rates were low, but with steeper decline, when atrophy rates reached levels characteristic of dementia [92]. These findings align with Stern’s model of CR [19], emphasizing the need to account for diagnostic status at both baseline and follow-up, as well as biomarker severity, when evaluating the effects of CR.

Several studies investigated the association between CR proxy variables and longitudinal changes in AD and related biomarkers. In middle-aged and older adults who did not have dementia, greater physical activity was frequently correlated to reduced brain atrophy over time, although findings remain inconsistent [58,101,102,103]. Higher levels of physical fitness and engagement in social activities had similarly been associated with less decline in white matter microstructural integrity [104,105]. By contrast, studies evaluating other CR proxies, such as cognitive activities, education, occupation, and literacy in relation to rates of change in AD biomarkers and structural measures, yielded mixed results. While some reports suggested that higher CR was associated with attenuated decline in CSF Aβ and hippocampal volume, other investigations found no relationship between CR proxies and changes in amyloid burden, medial temporal lobe atrophy, fluorodeoxyglucose (FDG) metabolism, or CSF tau and p-tau levels [106,107]. Among individuals with AD dementia, higher educational attainment was paradoxically associated with greater cortical thinning and larger decreases in cerebral blood flow over time [43,44,79,107].

Current findings suggested that higher levels of physical activity may help mitigate structural brain changes over time in non-demented populations, including reduced atrophy and preserved white matter microstructure [41,101,102,107]. In contrast, evidence for the influence of other CR proxies, residual variance approaches, and neurobiological indicators on longitudinal trajectories of AD biomarkers or brain structure and function remains limited and inconsistent. Notably, most available studies were constrained by relatively short follow-up periods for biomarker assessment (2–4 years), which limit the conclusions that could be drawn about long-term effects [108,109].

A further limitation of longitudinal CR research concerns the substantial temporal gap between the establishment of CR proxies and the onset of neuropathological change in older adulthood. Static, early-life indicators such as education or childhood cognitive performance may be separated from AD-related pathology by several decades, complicating causal interpretation [98]. These temporal lags raise the possibility of reverse causality, whereby lower early cognitive ability contributed to both reduced educational attainment and heightened vulnerability to later-life neuropathology, rather than education exerting a protective effect [110]. Moreover, many longitudinal cohorts begin observation after pathological processes were already underway, limiting the ability to disentangle the timing and directionality of associations between CR proxies, brain changes, and cognitive trajectories. Acknowledging these temporal and methodological constraints was essential for interpreting the effects of proxy-based CR within aging populations.

## 8. Summary

CR emerged as a central explanatory construct for understanding why individuals with comparable neuropathological burden exhibited divergent cognitive and clinical trajectories. Evidence synthesized from sociobehavioral, residual variance, and neurobiological studies consistently indicated that higher CR enhanced resilience to age- and disease-related brain changes, moderating both the level and rate of cognitive decline. Sociobehavioral proxies, such as educational level, occupational complexity, bilingualism, and engagement in cognitively enriching activities, captured the life-course experiences that fosterd the reserve capacity; however, they provided only indirect estimates of its underlying neural mechanisms. Complementing these traditional proxies, residual variance approaches offered a quantitatively precise method by identifying “better-than-expected” cognitive performance beyond that predicted from brain integrity or biomarker measures, thereby capturing the statistical expression of latent resilience. Longitudinal evidence further supported the view that CR was a dynamic rather than static construct with higher residual-based reserve predicting slower decline, delayed onset of MCI and AD, and superior functional outcomes over time.

Converging neuroimaging, electrophysiological, and genetic findings highlight the biological substrates through which CR exerts its effects, enhancing neural efficiency, network flexibility, and compensatory recruitment to maintain cognitive performance in the face of pathology. Nonetheless, variability in operational definitions, measurement frameworks, and analytical models continued to challenge comparability across studies. Future research should prioritize integrative, multimodal, and longitudinal designs that combine sociobehavioral proxies, residual variance metrics, and neurobiological indicators to elucidate the mechanisms through which reserve develops, adapts, and was maintained across the lifespan. Establishing unified and validated models of CR will be essential for translating the theoretical insights into practical interventions that strengthen cognitive resilience, delay clinical manifestation of dementia, and promote healthy cognitive aging. Ultimately, CR should be viewed not only as a theoretical moderator of brain–behavior relationships, but also as a modifiable target for preventive and therapeutic strategies in neurodegenerative diseases. Although many traditional CR proxies, such as education or childhood cognitive ability, were largely fixed in later life, several lifestyle and experiential factors remained meaningfully modifiable. Activities that involved the sustained cognitive engagement, physical exercise, and social participation could enhance adaptive neural processes and bolster compensatory capacity, even in older adulthood. These later-life influences did not alter structural reserve, but could strengthen functional resilience, supporting the view that CR retained the translational relevance, as a target for preventive and therapeutic strategies.

## Figures and Tables

**Table 1:** Summary of representative longitudinal and biomarker-informed studies examining CR.

Study	Design/Sample	CR Proxy	Covariates/Confounders Controlled	Key Findings	Key Limitations
Stern 2012 [15]	Narrative + empirical models; multiple cohorts	Education, IQ, occupation	Varied across cohorts; SES often partial; APOE rarely included	CR moderates clinical expression of AD pathology	Heterogeneous measures; limited biomarker integration
Vemuri et al., 2011 (ADNI) [21]	Longitudinal; *n* ≈ 300 CN/MCI/AD	Education, occupation	Age, sex, MRI, FDG-PET; no SES, no APOE	CR and biomarkers independently predict cognition	CR proxies limited; SES/education quality unmeasured
Soldan et al., 2017 (BIOCARD) [39]	20-year longitudinal; *n* ≈ 350 CN	Residual variance (memory)	Age, sex, MRI, CSF Aβ/tau; APOE included	Higher residual-CR predicts slower decline	Highly educated cohort; limited SES diversity
Franzmeier et al., 2017 [40]	fMRI connectivity; *n* ≈ 100 MCI	Education, connectivity	Age, sex, hippocampal volume; APOE included	Left frontal connectivity underlies CR	Small sample; connectivity measures variable
Gazes et al., 2023 [35]	5-year longitudinal; *n* = 254	IQ, education	Age, sex, cortical thickness, MD; no SES	CR and brain maintenance independently predict decline	Mid-SES sample; cognitive tests limited
DeJong et al., 2023 [41]	Population dMRI cohort; *n* = 4759	Structural connectivity	SES, vascular risks; no APOE	Connectivity buffers effect of atrophy on cognition	Cross-sectional cognition; dMRI quality variable
van Loenhoud et al., 2019 [25]	ADNI; *n* ≈ 500	Education	Age, sex, Aβ, tau; APOE included	Paradoxical faster decline in high-CR once symptomatic	CR paradox sensitive to model assumptions

This table synthesized key design features, CR proxies, confounder adjustment (including socioeconomic status and APOE ε4), principal findings, and methodological limitations across frequently cited studies. The comparison presented the substantial heterogeneity in CR operationalization and incomplete control of major confounders, which complicated the interpretation and comparability of results. Abbreviations: CN = cognitively normal; MCI = mild cognitive impairment; AD = Alzheimer’s disease; ADNI = Alzheimer’s Disease Neuroimaging Initiative; BIOCARD = Biomarkers of Cognitive Decline study; SES = socioeconomic status; APOE = apolipoprotein E; IQ = intelligence quotient; MRI = magnetic resonance imaging; FDG-PET = fluorodeoxyglucose positron emission tomography; CSF = cerebrospinal fluid; Aβ = amyloid-beta; fMRI = functional magnetic resonance imaging; dMRI = diffusion magnetic resonance imaging; MD = mean diffusivity; CR = cognitive reserve.

## Data Availability

The data supporting the findings of this study were available upon reasonable request from the corresponding author Y.C.Y. via email. Due to confidentiality agreements and institutional policies protecting sensitive patient information from the hospital where the study was conducted, the data cannot be made publicly available. Requests will be evaluated to ensure compliance with ethical and legal standards.
